# Microvascular Free Flap Reconstruction After Salvage Total Laryngectomy: Experience of the Verona University

**DOI:** 10.3390/jcm14207155

**Published:** 2025-10-10

**Authors:** Riccardo Nocini, Giulia Gobbo, Valerio Arietti, Gabriele Molteni, Luca Sacchetto, Giorgio Barbera, Gianluca Colapinto, Massimo Del Fabbro, Funda Goker

**Affiliations:** 1 Unit of Otorhinolaryngology, Head and Neck Department, Azienda Ospedaliera Universitaria Integrata, 37134 Verona, Italy; riccardo.nocini@aovr.veneto.it (R.N.); giulia.gobbo@univr.it (G.G.); luca.sacchetto@univr.it (L.S.); 2 Department of Medical and Surgical Sciences, Alma Mater Studiorum, University of Bologna, 40138 Bologna, Italy; 3 Otolaryngology and Audiology Unit, IRCCS Azienda Ospedaliero, Universitaria of Bologna, 40138 Bologna, Italy; 4 Maxillo Facial Surgery Unit, Head and Neck Department, University of Verona, 37134 Verona, Italy; giorgio.barbera@aovr.veneto.it; 5 Department of Biomedical, Surgical and Dental Sciences, Università Degli Studi di Milano, 20122 Milan, Italy; massimo.delfabbro@unimi.it (M.D.F.); funda.goker@unimi.it (F.G.); 6 Unit of Maxillofacial Surgery and Dentistry, Fondazione IRCCS Ca’ Granda Ospedale Maggiore Policlinico, 20122 Milan, Italy; 7 Department of Oral and Maxillofacial Surgery, Faculty of Dentistry, Istanbul Aydın University, 34295 Istanbul, Turkey

**Keywords:** head and neck cancer, laryngeal tumors, microvascular free flaps, total laryngectomy

## Abstract

**Objective:** This article evaluates the reconstructive potential and functional outcomes, as well as the risks and potential perioperative complications of using free flaps in patients with advanced-stage malignant laryngeal neoplasms who require salvage surgery and reconstruction. Additionally, it assesses the effectiveness of various flap harvesting and in-setting techniques, including the performance of microvascular anastomoses. **Materials and Methods:** This retrospective study included 13 male patients (age range 47–76 years) diagnosed with laryngeal neoplasms, who were referred to the Department of Otolaryngology at the University of Verona between 2017 and 2022. All patients underwent salvage total laryngectomy followed by concurrent reconstructive surgery utilizing microvascular flaps. Recovery of function (phonation) and incidence of complications were evaluated in a follow-up of at least three years. **Results:** Only one patient experienced necrotic failure of the microvascular free flap, probably due to post-op complications. The patient required revision on the 10th day after surgery and was reconstructed using a pedicled pectoralis major muscle flap. Two patients developed a pharyngocutaneous fistula. Other three patients had pharyngoesophageal stenosis, two experienced recurrence, and one patient passed away due to septic shock. All patients achieved satisfactory functional outcomes regarding vocalization, while complete oral intake was restored in eight patients. **Conclusions:** Considering the limited sample size, the findings suggest that microvascular flaps represent a feasible option for reconstructing advanced laryngeal tumors, though complication rate may still be considerable. Tailoring reconstructive approaches to individual patients may enhance surgical outcomes.

## 1. Introduction

In recent decades, the treatment of advanced laryngeal cancer has evolved significantly. Radiotherapy (RT) and chemoradiotherapy (CRT) have become standard care protocols aimed at organ preservation, focusing on maintaining patients’ natural speech and swallowing functions while providing survival rates comparable to primary surgical options. However, a substantial subset of patients (between 21% and 66%) eventually requires salvage total laryngectomy (STL) due to residual or recurrent cancer [1,2]. Fortunately, advances in rehabilitation techniques enable many of these patients to regain functional swallowing and speech, with some even achieving hands-free voicing. [3].

Salvage surgery represents the most effective option for durable disease control and for restoring the ability to feeding orally. However, it is technically challenging. This challenge arises from high perioperative morbidity and mortality rates, along with the generally poor prognosis associated with recurrent disease [4,5]. The complexities of tumor resection and defect reconstruction are exacerbated by CRT’s harmful effects on tissue microvasculature, which can lead to subintimal fibrosis, distortion of surgical planes, and a hypovascular, hypoxic tissue environment that complicates wound healing. These compromised tissues increase the risk of pharyngeal closure failure, with the development of pharyngocutaneous fistulas (PCF) reported to occur in up to 33% of cases [1,2,6].

To reduce the high incidence of PCF during salvage surgery, various reconstructive techniques have been developed, including salivary bypass tubes, secondary tracheoesophageal puncture, hyperbaric oxygen therapy, botulinum toxin injections, and negative pressure wound therapy [2]. One of the most extensively studied interventions involves the transplantation of vascularized tissue, such as regional or free flaps, into the irradiated area. Free flaps aid in revascularizing the neopharynx, promoting wound healing and mitigating postoperative complications like inflammation, fibrosis, and infection, which can increase the risk of fistula formation. These flaps can serve as onlay grafts that act as biological dressings to reinforce suture lines, patches for partial reconstruction of the pharyngeal lumen, or in circumferential reconstructions when substantial mucosal loss calls for full pharyngeal reconstruction [6].

In recent decades, microvascular free flaps have been extensively used for reconstruction procedures in head and neck cancer, proving similar or superior to pedicled flaps regarding versatility and clinical outcomes [7,8,9]. One study on 114 patients affected by cancer-related defects, who underwent a one-stage reconstructive procedure using either the pectoralis major myocutaneous flap or radial forearm-free flap reported a marked superiority of free flaps over pedicled flaps in improving speech performance [10]. The same study found a >50% post-op incidence of flap dehiscence in the pedicled flap, and a significant occurrence of atelectasis in the free flap [10]. Some recent systematic reviews and meta-analyses reported variable performance of free flaps compared to different types of pedicled flaps [11,12,13]. A meta-analysis by Tonsbeek et al. (2024) compared functional outcomes and complication rates of different techniques for surgical reconstruction of partial hypopharyngeal defects after total laryngectomy [13]. They could not find a technique superior to the others in all parameters evaluated and concluded that free flaps and pectoralis major myofascial flaps might be preferred for the type of reconstruction investigated. In general, these systematic reviews reported that microvascular free flaps were not inferior to pedicled flaps in terms of clinical outcomes, morbidity, recurrence rates and overall complications, while they may be associated with longer operative time and hospitalization [11,12,13].

In this single-center retrospective study, we reviewed the reconstructive surgical strategies used in salvage laryngectomy to address the challenges of impaired wound healing and pharyngoplasty breakdown, as well as to manage hypopharyngeal defects following STL in patients who did not achieve success with first-line curative CRT. Our primary objective was to evaluate the impact of these surgical approaches on the incidence of PCF, postoperative dysphagia or pharyngoesophageal stenosis (PES), and functional speech outcomes.

## 2. Materials and Methods

Twenty-seven patients underwent salvage total laryngectomy between 2017 and 2022 at the Head and Neck Department—Otorhinolaryngology Unit of the University Hospital of Verona, Italy. All patients enrolled in the study had previously undergone surgical or chemoradiotherapy treatments. Among the 27 patients, a pectoralis major pedicled flap was chosen for 14 of them, while 13 patients underwent reconstruction with a microvascular flap and were considered for this study. Microvascular flaps consisted of free temporal fascia flaps (n = 3 cases), anterolateral thigh (ALT) flaps (five cases), radial forearm free flaps (RFFF, three cases), and free latissimus dorsi flaps (two cases). All patients were male, confirming the gender predominance of laryngeal cancer in the population [14]. The average age was 64.8 ± 8.5 years (range 47–77 years). The main risk factors included smoking and alcoholism. Table 1 presents the preliminary data of the patients enrolled in the study, the reconstructive technique used, the nutrition type, and the complications encountered.

### 2.1. Design of the Study

This study was a retrospective, monocentric evaluation of patients who underwent STL at the Complex Operative Unit of Otolaryngology at the University of Verona from 2017 to 2022. Due to the retrospective nature of the study, no ethical approval was required. All the procedures performed in this study adhered to the ethical standards of the committee on human experimentation of the Institution, and in agreement with the Helsinki Declaration of 1975 as revised in 2013. The aim of the study was to assess clinical performance and the risks associated with using microvascular flaps in salvage laryngeal surgery.

At our institution, total laryngectomy (TL) is reserved for patients with advanced-stage disease (T3/T4 according to the Tumor, Nodes, Metastasis (TNM) classification of the American Joint Committee on Cancer (AJCC) [15]), while early-stage disease (T1/T2) is typically managed with laser microsurgery or open partial horizontal laryngectomy (OPHL). Nonsurgical treatment for early-stage disease, which adheres to an organ-preservation protocol, is offered less frequently. This practice explains the low percentage of salvage surgeries and prior CRT/RT) treatments at our center, resulting in a limited sample size for this study. Furthermore, patients undergoing salvage laryngectomy underwent the free flap reconstructive phase only if the examination of the involved region confirmed oncological radicality of the resection margins.

The inclusion criteria for this study were as follows:

- Prior radical or adjuvant CRT for laryngeal squamous cell carcinoma (LSCC).
- Tumor persistence, relapse, or chondroradionecrosis requiring STL.
- The need for surgical defect reconstruction with a flap.
- Treatment with curative intent.

The exclusion criteria were:

- Unresectable tumors that cannot be treated using laser or other minimally invasive techniques.
- Reconstructive techniques that utilize a pedicled flap.

These criteria ensure that the study focuses on cases requiring more extensive reconstructive procedures, such as microvascular flaps.

### 2.2. Outcome Variables

The study evaluated the impact of the surgical approach with free flap by assessing functional outcomes (speech, swallowing) and incidence of complications (pharyngocutaneous fistula, postoperative dysphagia, and any other complication). The recovery of swallowing and speech function was assessed by an ENT specialist (V.A, G.M.). The patient’s ability to swallow was based on the type of food consumed, whether solid or liquid, and calculates the time of the swallowing phase between one event and the next. Recovery of speech in patients with voice prostheses, was assessed by the VHI (voice handicap index), evaluating the patient’s ability to produce easily intelligible and comprehensible sounds.

## 3. Results

Of the 13 patients who underwent salvage total laryngectomy, 10 had total laryngectomy, two underwent total glosso-pharyngo-laryngectomy, and one patient received total pharyngo–laryngo-esophagectomy. All procedures were followed by reconstruction of the surgical defect using a microvascular free flap. Two surgeons performed the surgeries (R.N. and G.M.). In terms of reconstructive procedures, we utilized three free temporal fascia flaps, five anterolateral thigh flaps, three free radial forearm flaps, and two free latissimus dorsi flaps. The superficial temporal fascia flap was applied to support pharyngoplasty, similar to the anterolateral thigh flap, in patient number 5. In other cases, reconstructive flaps were employed in various configurations: tubed to fully restore hypopharyngeal continuity, as patches to address isolated pharyngeal defects, or in a chimeric configuration for extensive reconstructions involving the oropharynx and/or oral cavity.

The reconstructive configurations used (patch, tubed, onlay) are also reported in Table 1. Additionally, three patients had a salivary stent placed concurrently during the resection. Of these, two stents were sutured at the base of the tongue and one at the level of the upper esophageal sphincter. Three months postoperatively all stents were removed under general anesthesia.

In six treated cases involving extensive resection of the pharyngeal mucosa, it was necessary to completely or partially tube the microvascular flaps (three radial forearm free flaps and three anterolateral thigh flaps) to restore the continuity of the digestive tract. For partial pharyngeal reconstruction, either a nasogastric tube or a salivary stent was used as a guide. All pharyngoplasties were constructed in a T-shaped configuration using manual suturing. In 10 out of the 13 cases reviewed, microsurgical anastomoses were performed with the Zeiss Pentero microscope system. Surgical loupes were utilized for flap harvesting and in-setting. The 3D Video Telescope Operating Monitor (VITOM^®^) system (Karl Storz GmbH, Tuttlingen, Germany) was employed in only three cases for both microsurgical anastomoses and flap harvesting and in-setting. The anastomoses were primarily performed using an end-to-end technique, except for two patients (cases 9 and 10), where the venous anastomosis was performed using an end-to-side technique [16].

Among the 13 patients who underwent reconstruction with a microvascular free flap, there was only one case of flap failure, requiring revision on the 10th postoperative day and necessitating reconstruction with a pedicled pectoralis major muscle flap. The cause of flap failure was not identified but was considered unrelated to microvascular anastomosis and more likely due to post-op complications. Two patients developed fistulas, one of which was managed conservatively with compression dressings, while the other required surgical revision, revealing a dehiscence of the flap suture at the base of the tongue. Notably, only one patient experienced issues at the donor site, necessitating a skin graft to treat dehiscence at the flap harvesting site (RFFF).

From a functional standpoint, eight patients fully resumed oral feeding, while one patient continues to feed both orally and through a percutaneous endoscopic gastrectomy (PEG) tube. The remaining patients continued feeding through PEG, because of significant preoperative dysphagia, related to the effects of previous radiotherapy and one via jejunostomy due to severe hypopharyngeal stenosis. Among the oral feeding patients, two developed progressive dysphagia due to extrinsic compression of the hypopharynx by recurrent disease, needing PEG placement, with poor prognosis. One patient passed away in the weeks following surgery due to septic shock. Regarding speech rehabilitation, four patients were successfully treated with the placement of a voice prosthesis, and one patient was trained to use esophageal speech. Only one patient (patient n. 9) had the voice prosthesis placed concurrently with the main surgery, while in the other three patients, it was performed in secondary stage with blind technique, as described by Pighi et al. [17]. The only failure noted was in a patient who underwent a pharyngo–laryngo-esophagectomy (patient number 13). This patient experienced recurrent closure of the tracheoesophageal fistula after multiple placement attempts. The decision to insert a voice prosthesis was determined by the quality of the reconstruction, achieved by completely restoring the airway and digestive tract, the thickness of the walls of the reconstruction itself capable of supporting the prosthesis, the patient’s age, and the oncological condition, staging, and prognosis. Regarding the outcomes of the reconstructive techniques, Table 1 reports the main complications associated with each method used. Two patients developed a pharyngocutaneous fistula: one following a radial forearm free flap (RFFF) and the other after a tubulized ALT flap. Additionally, three patients presented with pharyngoesophageal stenosis—one with an RFFF, one with a tubulized ALT flap, and one with a temporalis fascia flap. Among these patients, only two required multiple esophageal dilations, with successful outcomes. One of these patients regained complete oral intake, while the other was limited to liquids only. The third patient was not eligible for further procedures due to poor overall health.

One patient treated with RFFF flap experienced late surgical complications. All four patients who underwent tracheoesophageal puncture (TEP) achieved intelligible tracheoesophageal speech, including three secondary and one primary TEP. One of them demonstrated a good-quality esophageal voice. The outcomes and complications reported by type of reconstruction configuration are presented in Supplementary Table S1. In spite of the high proportion of complications, all of them were managed successfully.

The nutritional status, which was adequate for the initial surgery for all patients (Table 1), was maintained through various procedures such as PEG, without ever reaching a state of malnutrition that could compromise the flap’s viability or contribute to its failure.

## 4. Discussion

The optimal reconstructive approach following salvage laryngeal surgery is still a matter of debate, with no consensus reached in the current literature [6,9,11,12,13]. Nevertheless, it is widely recognized that minimizing local complications resulting from previous radiation therapy (RT) is essential. This goal can be achieved using regional pedicled flaps, primarily the pectoralis major myocutaneous flap or free flaps that require microvascular anastomosis. In many hospitals and universities, consistent with the scientific literature, the use of the pectoralis major flap for the reconstructive phase after salvage laryngectomy represents the first choice, or very often the only option, depending on the patient’s clinical condition. Our experience as an institution in terms of outcomes with pectoralis major flap is in line with that of other institutions and the pertinent literature. In the present study, we focused our attention on the impact of the reconstructive phase with free flaps, which are not used often, because patients undergoing salvage laryngectomy often have a history of chemoradiotherapy. The latter produces relevant side effects, leading to a depletion of stem cell reserves, a reduction in vascularization and tissue fibrosis. This makes the survival of free flaps more challenging, with higher failure rates, compared to locoregional flaps such as the pectoralis major flap, where the vessels are not subjected to the trauma of anastomoses and have not suffered from actinic damage. Furthermore, a pedicled flap like the local pectoralis major flap can always be performed in case of failure of the free flap. The findings of this study suggest that microvascular free flaps represent a feasible option for surgical reconstruction of advanced laryngeal tumors, even if the complication rate may not be negligible. The choice of analyzing a single cohort and the limited sample size may represent relevant limitations for the generalizability of the results, which should be interpreted cautiously and need to be confirmed by prospective comparative studies with a wider sample.

Although using a free flap carries a higher risk of early and late complications related to the anastomotic procedure compared to pedicled flap, it allows for more customized reconstruction tailored to the specific characteristics of the patient’s defect, such as size, thickness, and necessary tissue type. Additionally, implementing a microvascular flap as the primary reconstructive strategy preserves the option of using a pedicled flap as a salvage procedure in case of free flap failure.

Salvage laryngectomy may result in total or partial pharyngeal defects, which can be categorized into three types: (a) defects with enough pharyngeal mucosa for primary closure; (b) defects with a posterior strip of pharyngeal mucosa that is insufficient; and (c) complete circumferential defects, where a 360-degree segment of pharyngeal mucosa is absent. Hui et al. demonstrated that primary closure can lead to acceptable swallowing outcomes when at least 1.5 cm of relaxed or 2.5 cm of stretched pharyngeal mucosa remains [18]. For defects where a strip of pharyngeal mucosa remains after ablative surgery, a patch reconstruction is performed to recreate the anterior pharyngeal wall. In these cases, the flap is sutured to the remaining mucosal strip, resulting in a horseshoe-shaped neopharynx. For circumferential defects, tubed flap reconstruction is necessary to restore the continuity of the pharynx. Several reconstructive options are available for these defects, utilizing both pedicled and free flaps [19].

Fasciocutaneous flaps are crucial for pharyngeal reconstruction. Among these, the RFFF and the ALT flap are the most commonly used and have gained significant popularity [5,20]. Moreover, the superficial temporal fascia flap, while less commonly used, has demonstrated promising results [19].

The RFFF is the most versatile flap, ideal for use in the oral cavity, pharynx, and cervical esophagus [21]. This flap is easy to harvest and has several advantages, including a long pedicle with well-sized vessels, a thin and highly pliable structure, and minimal long-term dimensional shrinkage. It can be harvested as a fascia-only flap to serve as a pharyngeal interposition graft for reinforcing primary pharyngeal closure. Alternatively, it can be harvested with a skin paddle to function as a patch graft or used for tubed pharyngeal reconstruction [19,22]. While it may seem that primary closure—using only pharyngeal tissue to create the pharyngoplasty and neopharynx—would lead to better swallowing outcomes, assuming no stricture develops, and result in lower rates of pharyngocutaneous fistula, Yeh et al. found a significantly higher rate of fistulas in the primary closure group (50%) compared to the free flap patch graft group (18%) [19]. According to the literature review by Piazza et al., the rate of PCF ranges from 2% to 53%, with a mean of 20%. Distal stenosis occurs in 0% to 36% of cases, with a mean of 11% [20]. In most cases, PCFs after radial forearm free flap reconstructions heal on their own without requiring additional surgical interventions [20]. After a total laryngectomy, preventing stenosis or stricture formation is essential for preserving the ability to swallow [19]. Withrow et al. demonstrated that using a free flap as a patch graft for reconstruction after salvage laryngectomy results in lower rates of stricture formation (18% compared to 25%) and less feeding tube dependence (23% versus 45%) when compared to primary closure [23]. In the study by Azizzadeh et al., the rate of oral intake improved to 85% after pharyngoesophageal reconstruction using RFFF [22]. In our series, only one patient who underwent RFFF reconstruction (33.34%) was able to resume oral intake with a liquid diet while also depending on a PEG for nutritional support.

The ALT flap is one of the most used fasciocutaneous flaps in salvage laryngeal surgery, thanks to its versatility and advantageous properties. It offers a significant amount of pliable skin, making it suitable for reconstructing circumferential pharyngeal defects of any length [20,21]. The ALT flap demonstrates greater variability in its vascular supply and thickness compared to the RFFF, and this variability can be influenced by the patient’s body habitus. This characteristic allows for the design of two separate skin paddles. These paddles can either be based on different perforators or connected by a deepithelialized bridge made of subcutaneous tissue. This design facilitates simultaneous reconstruction of the pharyngoesophageal lumen along with any related cervical skin defects [24,25]. Additionally, the external skin paddle serves as an effective monitoring tool for the buried portion of the flap, without increasing the risk of postoperative pharyngocutaneous fistulas (PCFs) or stenosis [26]. The ALT flap can incorporate the vastus lateralis muscle or be raised as an adipofascial flap to serve as an onlay graft, providing additional reinforcement to the primary closure line [19]. Recent studies have compared the outcomes of two types of fasciocutaneous flaps. The rate of pharyngocutaneous fistula following an ALT flap reconstruction is lower than that observed with a RFFF. This difference may be due to the significant fascial component provided by the fascia lata, which reinforces the primary suture line with a strong secondary layer. The lower incidence of PCF is also linked to a reduced occurrence of distal strictures [27]. The chimeric structure of the ALT flap is especially beneficial in complex reconstructions, where it is crucial to both protect the pharyngeal suture line and cover major cervical vessels [20].

Although existing literature indicates the ALT flap is associated with a lower risk of PCF and PES, Lane et al. in 2024 reported that the overall fistula rate did not significantly differ between vascularized tissue augmentation using muscle and that without muscle [28]. Additionally, there was no significant difference in the rate of fistulas that required reoperation between the two methods [2].

Both RFFF and ALT techniques utilize Montgomery salivary bypass stents to reduce the likelihood of fistula formation and stenosis. This is achieved by minimizing the exposure of the anastomotic suture line to saliva and providing support to the reconstruction during the early healing phase [29,30]. Despite their potential benefits, there is still considerable disagreement regarding their effectiveness [21]. A recent review conducted by Lopez et al. examined the use of salivary bypass tubes in fasciocutaneous flaps for hypopharyngeal reconstruction. The review reported an overall fistula rate of 9%. Specifically, the rate was 16% for RFFF and 3% for ALT. Additionally, the stricture rate was observed to be 5%, with 8% for RFFF and 3% for ALT [31]. In our cohort, only one of the three patients who used a salivary stent developed a salivary fistula, which required surgical intervention. There were no cases of pharyngoesophageal stricture associated with the use of a salivary stent. In 2021, Crosetti et al. introduced the “fistula zero protocol” to reduce the incidence of postoperative complications, specifically PCF, following transoral surgery with a salivary stent [32]. Among patients who had not received prior radiotherapy or chemotherapy, PCF occurred in only one case (1.3%). In contrast, there were three cases (3.9%) of blind fistulas, two of which occurred in patients who had been pretreated.

The temporoparietal fascia flap (TFFF) offers a viable alternative to the previously discussed free flaps in the context of salvage total laryngectomy. Studies have demonstrated that using the TFFF is linked to a decreased incidence of PCFs and a greater likelihood of spontaneous healing compared to primary closure [2]. In the case series by Pellini et al., a lower incidence of PCF was observed, occurring in only two patients (14%); however, this finding was not statistically significant [33]. In our sample, the flap demonstrated high effectiveness, with no surgical or functional complications, and there were no instances of PCF or PES development. The temporoparietal fascia is a thin and flexible flap that can be harvested either as a free flap or as a pedicled flap, both of which are based on the superficial temporal artery and vein. This versatility allows it to be used as an overlay flap with minimal donor-site complications.

The flap is then transposed to the neck, where it is utilized to cover the pharyngeal mucosa. It is secured superiorly to the base of the tongue, laterally to the prevertebral fascia, and inferiorly to the tracheoesophageal septum. The temporoparietal fascia acts as a physical barrier, effectively preventing the formation of pharyngocutaneous fistulas [34]. In our case series, TFFF has proven to be a very effective flap, with a low incidence of surgical complications and no formation of PCF or PES.

Chimeric flaps are usually used for more complex resections that extend beyond the larynx and involve other anatomical areas. These procedures carry a significantly higher risk of complications, such as fistula formation and wound dehiscence. As a result, they often require the collaboration of two surgical teams, made up of highly trained surgeons who specialize in their respective fields, to ensure the best possible outcomes.

In addition to the single cohort design and the limited sample size, one of the limitations of this study is represented by the absence of a standardized quantitative tool for the assessment of voice and swallowing outcomes. Although the assessment of these functions is performed by experienced professionals, assessing the quality of recovery may be influenced by individual judgment. In addition, heterogeneity of the features of patients treated with free flaps might be seen as a further limitation. Conversely, this might represent a strong point because such flaps proved to be versatile in choosing the reconstructive phase, as they were easy to adapt and personalize the surgical procedure to the individual patient.

## 5. Conclusions

Salvage total laryngectomy is a complex surgical situation that carries a significant risk of postoperative and long-term complications. This is primarily due to chronic changes caused by radiation therapy, which severely impair tissue healing. Currently, no single surgical reconstruction technique for pharyngolaryngeal defects is suitable for all patients. In addition, scientific literature shows that different techniques are related to different advantages and drawbacks and the results in terms of effectiveness, morbidity, operative times, adverse effects and complication rate are often unpredictable as they may depend on a number of factors (patient-related, surgical protocol-related, and operator-related). Using a free flap as the first-line reconstructive option presents its own challenges and requires careful postoperative monitoring to ensure proper integration of the tissue at the recipient site. However, this approach allows for the option of using a pedicled flap as a backup solution in case the primary reconstruction fails.

The main advantage of free flap reconstruction is its customization potential. This begins with preoperative planning, flap selection, and in-setting, all with the goal of restoring continuity of the upper digestive tract and optimizing prosthetic phonation as much as possible. No universally preferred free or pedicled flap exists; even among well-established surgical techniques, multiple reconstructive options may be available. Therefore, decision-making should be tailored on a case-by-case basis to achieve the best possible surgical outcome for each patient.

## Figures and Tables

**Table 1:** Characteristics of the selected patients, their treatment and outcomes.

N.	Sex	Age	Surgery	Flap Type (Reconstruction Configuration)	Anastomosis	Tep	Nutrition	Complication/Treatment	Failure
1	M	76	Total laryngectomy	Temporalis fascia (onlay)	A: T-T superficial temporalis artery—superiori thyroid artery V: T-T temporalis vein—Collateral vein of the internal jugular vein	No	Oral intake	No	No
2	M	59	Total pharyngolaryngectomy	ALT (tubed)	A: T-T descending branch of circumflex lateral femoral artery—superior thyroid artery V: T-T comitant vein—thyroid-lingual trunk	No	Digiunostomy	Severe PES	No
3	M	47	Total laryngectomy + emipharyngectomy	RFFF (patch)	A: T-T radial artery—superior thyroid artery V: T-T cephalic vein—thyroid-lingual trunk	No	PEG + oral intake (only liquids)	PES/esophageal balloon dilatation	No
4	M	74	Total pharyngolaryngectomy	ALT (tubed)	A: T-T descending branch of circumflex lateral femoral artery—superior thyroid artery V: T-T comitant vein—thyroid-lingual trunk	No	Oral intake	PCF/closed	No
5	M	64	Total laryngectomy	ALT (onlay)	A: T-T descending branch of circumflex lateral femoral artery—lingual artery V: T-T comitant vein—thyroid-lingual trunk	Yes (2)	Oral intake	No	No
6	M	61	Total glosso-pharyngo-laryngectomy	Latissimus dorsi and serratus	A: T-T the subscapular artery—transverse cervical artery V: T-T subscapular vein—external jugular vein	No	Oral intake	Recurrence/PEG positioning	No
7	M	60	Total laryngectomy	Temporalis fascia (onlay)	A: T-T superficial temporalis artery—superiori thyroid artery V: T-T temporalis vein—thyroid-lingual trunk	Yes (2)	PEG	No	No
8	M	72	Total laryngectomy	Temporalis fascia (onlay) + Salivary stent	A: T-T superficial temporalis artery—superiori thyroid artery V: T-T temporalis vein—thyroid-lingual trunk	Yes (2)	Oral Intake	Pharyngeal recurrence/PEG positioning	No
9	M	77	Total pharyngolaryngectomy	RFFF (tubed) + Salivary stent	A: T-T radial artery—facial artery V: T-L comitant vein—internal jugular vein	Yes (1)	PEG	PCF/closed	No
10	M	70	Total glosso-pharyngo-laryngectomy	Latissimus dorsi and serratus	A: T-T subscapular artery—superior thyroid artery V: T-L subscapular vein—internal jugular vein	No	Oral intake	No	No
11	M	63	Total pharyngolaryngectomy	RFFF (tubed) + Salivary stent	A: T-T radial artery—superior thyroid artery V: T-T cephalic-comitant vein—thyroid-lingual trunk	No	PEG	Delayed flap necrosis/pedicle PMF harvesting + PEG positioning	Yes
12	M	60	Total laryngectomy + oropharyngectomy + emiglossectomy and emipharyngectomy	ALT (patch)	A: T-T descending branch of circumflex lateral femoral artery—superior thyroid artery V: T-T-comitant vein—thyroid-lingual trunk	No	Oral intake	Septic shock	No
13	M	60	Total pharyngo–laryngo-esophagectomy	ALT (tubed)	A: T-T descending branch of circumflex lateral femoral artery—facial artery V: T-T-comitant vein—branch internal jugular vein	Multiple failures	Oral intake	PES/esophageal balloon dilatation	No

ALT: anterolateral thigh; TEP: tracheoesophageal puncture; RFFF: radial forearm free flap; T-T: Terminal to terminal anastomosis; T-L terminal to lateral anastomosis; PCF: pharyngocutaneous fistula; PEG: percutaneous endoscopic gastrectomy; PES: pharyngoesophageal stenosis; PMF: pectoralis major muscle flap.

## Data Availability

The original contributions presented in this study are included in the article. Further inquiries can be directed to the corresponding author.

## Supplementary Materials

The following supporting information can be downloaded at: https://www.mdpi.com/article/10.3390/jcm14207155/s1, Table S1: Outcomes per reconstruction configuration.
