# Supplementary material for: Microvascular Free Flap Reconstruction After Salvage Total Laryngectomy: Experience of the Verona University
Source: J Clin Med. 2025 Oct 10;14(20):7155. doi: 10.3390/jcm14207155 (PMC12564040; doi:10.3390/jcm14207155)
Supplement: Supplementary file 1 [file jcm-14-07155-s001.zip › jcm-3879013-supplementary.pdf]

Supplementary Table S1. Outcomes per reconstruction configuration.

Reconstruction configuration	Number of cases	TEP (No / yes)	Nutrition (Oral intake / PEG-other)	Complications (No / yes)	Failure (No / yes)
Tubed	5	3 / 2	2 / 3	2 / 3 (2 PCF, 1 PES)	4 / 1
Patch	2	2 / 0	1 / 1	0 / 2 (1 PES, 1 SS)	2 / 0
Onlay	4	1 / 3	3 / 1	3 / 1 (recurrence)	4 / 0
Chimeric	2	2 / 0	2 / 0	1 / 1 (recurrence)	2 / 0
Total	13	8 / 5	8 / 5	6 / 7	12 / 1

TEP: tracheo-esophageal puncture; PEG: percutaneous endoscopic gastrectomy; PCF: pharyngo-cutaneous fistula; PES: pharyngo-esophageal stenosis; SS: septic shock.
